# Smartwatches in healthcare medicine: assistance and monitoring; a scoping review

**DOI:** 10.1186/s12911-023-02350-w

**Published:** 2023-11-03

**Authors:** Mohsen Masoumian Hosseini, Seyedeh Toktam Masoumian Hosseini, Karim Qayumi, Shahriar Hosseinzadeh, Seyedeh Saba Sajadi Tabar

**Affiliations:** 1 Department of E-Learning in Medical Sciences, Tehran University of Medical Sciences, Tehran, Iran; 2 CyberPatient Research Affiliate, Interactive Health International, Department of the surgery, University of British Columbia, Vancouver, Canada; 3 Department of Nursing, School of Nursing and Midwifery, Torbat Heydariyeh University of Medical Sciences, Torbat Heydariyeh, Iran; 4 Professor at Department of Surgery, University of British Columbia, Vancouver, Canada; 5 CyberPatient Research Coordinator, Interactive Health International, Department of Surgery, University of British Columbia, Vancouver, Canada; 6 Universal Scientific Education and Research Network (USERN), Tehran, Iran

**Keywords:** Smartwatches, Wearable devices, Healthcare, Monitoring, AI-assistance, Validations

## Abstract

Smartwatches have become increasingly popular in recent times because of their capacity to track different health indicators, including heart rate, patterns of sleep, and physical movements. This scoping review aims to explore the utilisation of smartwatches within the healthcare sector. According to Arksey and O'Malley's methodology, an organised search was performed in PubMed/Medline, Scopus, Embase, Web of Science, ERIC and Google Scholar. In our search strategy, 761 articles were returned. The exclusion/inclusion criteria were applied. Finally, 35 articles were selected for extracting data. These included six studies on stress monitoring, six on movement disorders, three on sleep tracking, three on blood pressure, two on heart disease, six on covid pandemic, three on safety and six on validation. The use of smartwatches has been found to be effective in diagnosing the symptoms of various diseases. In particular, smartwatches have shown promise in detecting heart diseases, movement disorders, and even early signs of COVID-19. Nevertheless, it should be emphasised that there is an ongoing discussion concerning the reliability of smartwatch diagnoses within healthcare systems. Despite the potential advantages offered by utilising smartwatches for disease detection, it is imperative to approach their data interpretation with prudence. The discrepancies in detection between smartwatches and their algorithms have important implications for healthcare use. The accuracy and reliability of the algorithms used are crucial, as well as high accuracy in detecting changes in health status by the smartwatches themselves. This calls for the development of medical watches and the creation of AI-hospital assistants. These assistants will be designed to help with patient monitoring, appointment scheduling, and medication management tasks. They can educate patients and answer common questions, freeing healthcare providers to focus on more complex tasks.

## Introduction

In the mobile technology market, smartwatches are experiencing tremendous growth as wearables and medical devices converge to monitor personal health in real-time, including cardiovascular health measures [1, 2]. Consumers can now access a personalised medical data report through these devices, which could prove useful in preventing and treating diseases [3]. The rise of smartwatches has empowered numerous patients to actively engage in self-care and advocate for the health of others. With the increasing prevalence of these individuals, known as 'e-patients', it is imperative for medical institutions to adequately educate their students on effectively collaborating with and providing healthcare services to them. Failing to do so could potentially jeopardise patients' lives [4]. Similar to the access to online tools that smartwatches provide for medical schools and students, access to e-patients is also increasing. Mobile decision support tools, for example, use simple graphics to communicate calculator results (such as the Framingham Risk Score) in a way that is relevant to both the learner and the patient [5].

The Framingham Risk Score is an algorithm designed to assess the probability of developing cardiovascular disease over a period of 10 years [6]. Initially focused on coronary heart disease, it was expanded in 2008 to encompass cerebrovascular events, peripheral artery disease, and heart failure. Considering variables such as age, sex, blood pressure levels, cholesterol levels, smoking status, and diabetes status, this risk score applies specifically to individuals aged between 30–79 without any previous history of CVD [7]. There are many apps explicitly aimed at patients, including simple medical calculators, e.g. BMI calculators, risk calculators, medication reminders, pharmacy finders and even diagnostic tools [8]. As many patients are unable to communicate their condition to their companions or medical staff, these smart tools will help caregivers be more alert and apply more appropriate comfort measures. The notion of comfort measures has gained significant attention in both nursing and medical literature, reflecting a systematic approach that entails proactive engagement as well as thoughtful restraint [9]. These measures can range from the simplest to more intricate interventions, all serving to provide supportive care. Importantly, they are applicable across various healthcare settings and warrant careful consideration within the clinical management of patients burdened with multiple chronic comorbidities [9].

The FDA has approved clinical smartwatches for detecting medically significant events and recording and processing data [10]. There are no approved directly addressed smartwatches in the European Union (EU). However, to comply with EU regulations, smartwatch manufacturers must navigate through various frameworks such as the Radio Equipment Directive 2014/53/EU and Medical Device Regulation 2017/745. These regulations emphasize safety standards and touch upon essential aspects like electromagnetic compatibility and efficient radio spectrum utilization [11, 12].

Smartwatches often come with advanced features like a heart rate monitor. These high-tech devices utilize a technique called photoplethysmography to accurately measure the user's heart rate. By utilizing light beams and specialized sensors on the smartwatch, changes in blood volume flowing through the wrist can be precisely quantified. This process generates a PPG waveform that provides valuable data for determining an individual's heart rate [13, 14]. One instance where this technology proves beneficial is when an epilepsy patient wears a smartwatch equipped with an artificial intelligence (AI) algorithm capable of identifying high-risk seizures and initiating human assistance promptly [15]. This particular smartwatch provides physiological data to monitor vital signs and issues alerts that help save lives, as attending a seizure is associated with a lower rate of severe injury and death [15]. Other wearable devices run algorithms that detect conditions such as atrial fibrillation or medically necessary conditions where early intervention can positively impact the quality of life. Researchers have shown that wearable biomarkers are more accurate at predicting blood and urine measurements than two vital signs in the clinic. They can provide clinicians with predictions from thousands of measurements in a fraction of the time it takes to measure a single vital sign [13]. It is important to note that these smartwatches are based on algorithmic interpretations of clinical outcomes, and their predictive power ultimately depends on the support and strength of their algorithms [16, 17]. In this review, we discuss the applications of these wearable devices to patient health and HealthWorks' educational initiatives. This article seeks to answer several questions, including whether smartwatches can be trusted for clinical measurements and what might facilitate the development of trust in these devices.

## Method

### Reason for scoping review

A scoping review was conducted since AI and machine learning algorithms are emerging technologies applied to smartwatches. Additionally, scoping reviews are helpful when attempting to evaluate the effectiveness of large-scale or emerging research [18].

Scoping reviews aim to identify and map relevant evidence on a topic, field, context or question that meets predetermined inclusion criteria [19, 20]. They provide important insights into the characteristics of a body of evidence and can highlight knowledge gaps for subsequent syntheses. Unlike traditional systematic reviews, scoping reviews have broader questions and may include multiple types of evidence. Scoping reviews are useful for obtaining a comprehensive overview of the evidence and identifying gaps in the existing literature without the need for methodological judgement or risk of bias assessment [21]. They differ from evidence maps, which present the results of a systematic search in searchable databases to identify knowledge gaps and future research needs. Scoping reviews also differ from other forms of evidence synthesis. Scoping reviews do not make recommendations for clinical practise and often do not assess methodological quality or risk of bias in studies. The PRISMA ScR is a new approach to reporting scoping reviews based on the popular PRISMA statement and checklist [22].

A scoping review can collect different types of evidence from different areas, including both empirical and non-empirical sources [23]. This type of review is suitable for investigating, identifying, presenting, reporting, or discussing features or concepts across a wide range of evidence sources [24]. Scoping reviews are particularly useful when comparing measures is neither practical nor feasible due to cost or time constraints. Although they often involve reviews of numerous sources, these reviews do not expect or allow for statistical pooling, formal risk assessment, or quality assessment [25].

### Search strategy

This study's methodology was based on the scoping review methodology that was developed by Arksey and O'Malley [20] and used the methodological enhancement suggested by Levac et al. (2010) [26]. As outlined in this framework, scoping reviews have six stages: (1) identifying a research question; (2) identifying relevant studies; (3) selecting studies; (4) charting the data; (5) collating, summarizing and reporting the results and (6) consulting with stakeholders.

### Stage 1: Identifying the research question

In consultation with the research team and key stakeholders, the overall main research question developed is: ‘Whether all smartwatches can be trusted for clinical measurements and what might facilitate the development of trust in these devices?’.

### Stage 2: Identifying relevant studies

#### Search strategy and information sources

First step in identifying articles related to this topic was to conduct a limited search of PubMed/Medline, SCOPUS, Embase, Web of Sciences, and ERIC. For the development of a comprehensive search strategy, the text words contained in the titles of relevant articles, as well as the index terms utilized to describe the articles, were analyzed (see Table 1). Depending on the database and/or information source involved, the search strategy was tailored to include all keywords and index terms identified. Additional studies were screened from the reference list of all included sources of evidence. We also searched a variety of grey literature sources in order to ensure that all relevant information was obtained. The review team searched relevant grey literature databases (such as Grey Literature Report, Google Scholar, OpenGrey, and Web of Science Conference Proceedings) for studies, reports, and conference abstracts of interest to the topic. A Research librarian developed the search strategy and revised it following input from stakeholders. In order to prevent bias, the research team blinded the stakeholders to the original search strategy that was developed. The search papers focused only on English-language studies. Considering that smartwatch medical applications have only been introduced to the market recently, the search period was restricted to the period following 2017. After conducting the search, all citations identified were gathered into an EndNote 8 database and duplicates were removed.

**Table 1:** Database search strategy

Search strategy and queries							
(smartwatch^*^ OR wristband^*^ OR “fitness-bound” OR “wireless watch^*^” OR “Wearable movement sensors”) AND (healthcare OR “health medicine” OR telemedicine OR medical OR clinical OR medicine OR health OR fitness OR healthiness OR wellness OR soundness OR validat^*^ OR assessment^*^ OR reliabl*)							
**Database**	**PubMed**	**Medline**	**Scopus**	**Embase**	**Web Of Science**	**ERIC**	**Google scholar**
**Article**	107	28	196	191	132	56	34
**English**	105	28	191	191	129	56	34
**Total**	**734**						

This table presents the search strategy for specific keywords in different databases. This column provides an overview of the steps and techniques used to retrieve relevant information from each database based on the keywords organised

### Stage 3: Study selection

#### Inclusion/exclusion criteria

- Types of research: any type of study design, in addition to randomized controlled trials (RCT and CT), controlled clinical trials, case studies, correlational studies, longitudinal studies, experimental studies, and quasi-experimental studies. There were no limitations regarding the study design or geographic location.
- Types of Participants: All Participants that deal with the healthcare system
- Types of interventions: The study focused on smartwatches, wristband, fitness-bound, wireless watch, Wearable movement sensors
- The studies included focused on any of the following areas: (a) development; (b) implementation; (c) evaluation; or (d) comparative validation of such measures.
- Types of outcomes: satisfaction, knowledge, skills, attitudes, and behaviours were the outcomes of interest.

**Fig. 1:**
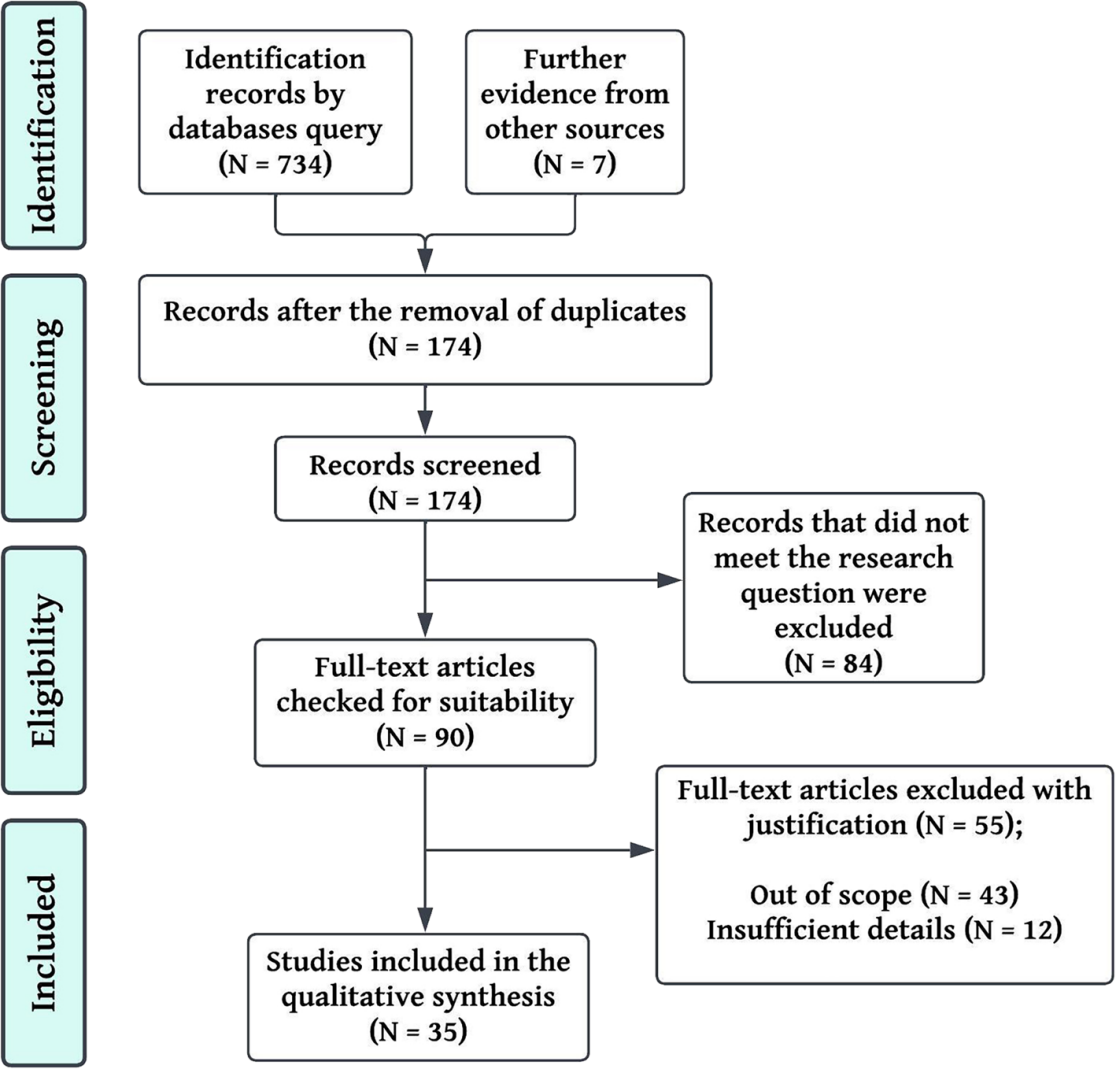
An overview of the article selection process according to ScR-PRISMA

Two screening stages took place in the review process: a review of the title and abstract, followed by an assessment of the complete text. The initial phase involved M.M.H and T.M.H, who independently scrutinized all obtained citations to determine if they met a set of minimum inclusion criteria. In order to ensure that the criteria were robust enough to capture any articles relating to smartwatches, a sample of abstracts was tested prior to undertaking the abstract review. The full-text review included any articles that either or both reviewers considered to be relevant. The full-text articles were then independently assessed by both investigators to determine if they fulfilled the inclusion and exclusion criteria. A Cohen's κ coefficient of the agreement was calculated both at the title and abstract review phase and at the full article review phase in order to determine the inter-rater agreement. In case of disagreements regarding study eligibility at the full-text review stage, any further disagreements were resolved through discussion with a third investigator until consensus was achieved. In the final scoping review, the search results and the inclusion process were comprehensively reported along with a flow diagram reflecting Preferred Reporting Items for Systematic Reviews and Meta-analyses extension for scoping reviews (PRISMA-ScR) (Fig. 1).

### Stage 4: Data collection

Data extraction was performed using the PRISMA-ScR Checklist, which consists of 22 items developed by IBJ [27]. To ensure that the form captured the information accurately, the form was reviewed by the research team and pretested by all reviewers before implementation. Studies characteristics that were extracted included to publication year, publication type (eg, original research or review type), study design, country, participants' population characteristics, intervention setting, description of quality indicators including definition, numerator, dominator, psychometrics of the indicators (face validity, reliability, construct validity, risk adjustment). The data abstraction process was conducted in duplicate by two reviewers who independently extracted the data from all included studies.

### Stage 5: Data summary and synthesis of results

Quality assessment is not an integral part of scoping reviews, but in this review, the quality checklist developed in BEME guide 11 was used to assess quality [28]. Based on the provided checklist, excellent quality studies met eight or more of the established criteria. Studies deemed moderate quality fulfilled six or seven indicators, while studies with low quality met five or fewer criteria. Each study was assigned either a 0 or 1 for each index measurement. Studies that met the index received a positive score, whereas studies that did not meet the index or were vaguely described received a zero score. Using the IBJ checklist as a guide, selected articles were analyzed and themes were extracted.

### Stage 6: Consultation

There is a suggestion by Levac et al. that the consultation stage provides opportunities for stakeholder involvement, which may provide insights beyond those that have been described in the literature. An integral part of the study's healthcare-centred approach was the involvement of stakeholders, including a patient partner who served as both a consultant and knowledge user throughout the study.

## Result

### Descriptive

**Fig. 2:**
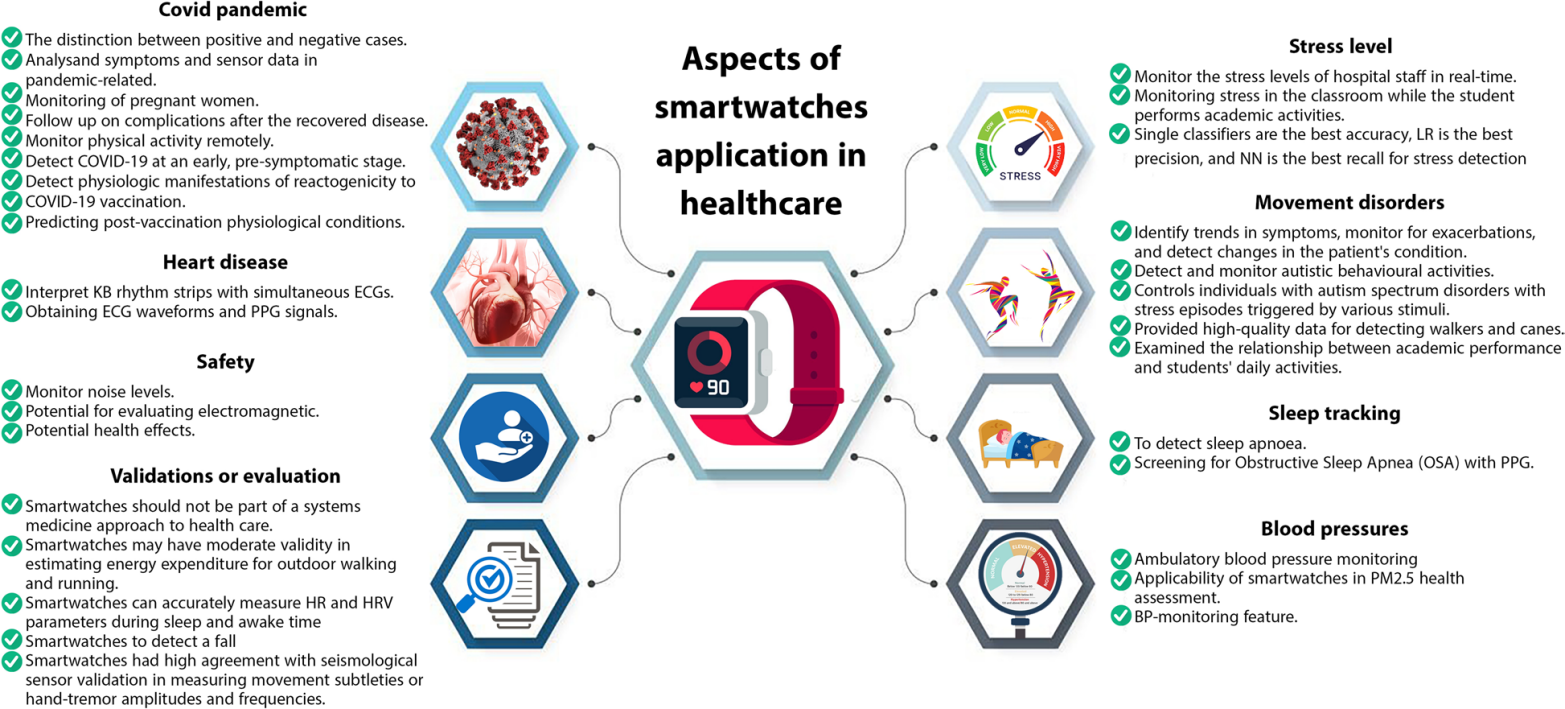
A graphical representation of the distribution and classification of studies based on the use of smartwatches

**Fig. 3:**
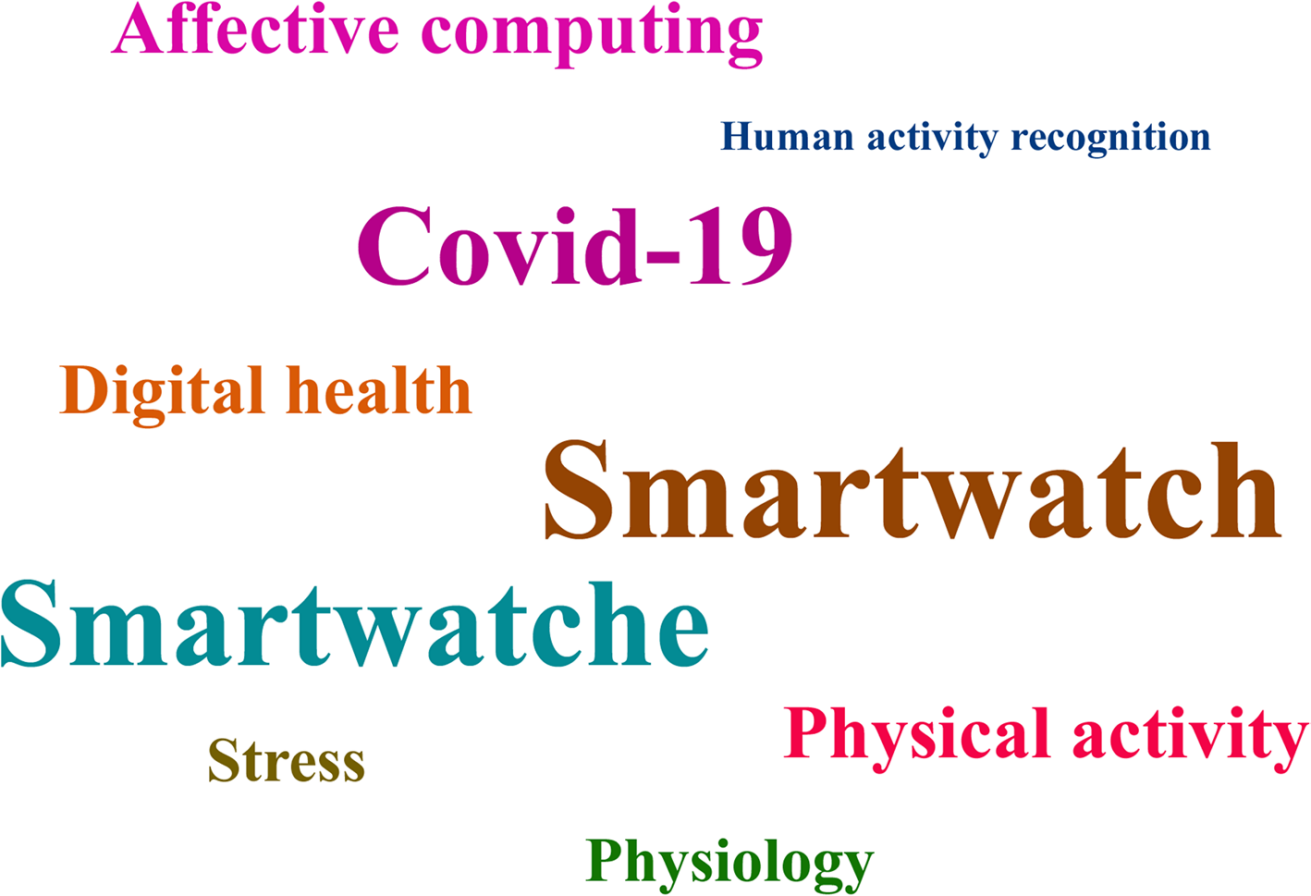
Keyword analysis of studies

Following the articles' quality assessment, 32 studies were analysed for data extraction. The majority of studies were conducted in the United State America (USA) (N = 10), three in China, three in Taiwan, three in United Kingdom (UK), in countries including Finland, Germany, Norway and Spain, each one two studies, and one study in the rest (Greece, Switzerland, Slovenia, Canada, Australia, Belgium, Brazil and France). Thirteen of the studies were experimental, four were cohort studies, and one study of each of the designs (longitudinal cohort study design, longitudinal observational study, multicenter observational study, randomized controlled trial (RCT), randomized, accelerometer data, combined method, feasibility study, observational study, prospective, nonrandomized, and adjudicator-blinded study, Prospective, single-arm, cross-sectional study, prospective comparative and prospective study). Three studies did not have a specific design due to the nature of the research, and one study was a systematic review (Table 2). As shown in Table 2, the geographical distribution of the studies, the purpose, the outcome and the (Table 2) number of participants are listed with a description of the type of study. In this review, the text was categorised according to the themes addressed in the studies, e.g. methodology, results and impact. We also identified the main themes and patterns that emerged from the analysis. This helped researchers understand the study's impact. These included six studies on stress monitoring, six on movement disorders, three on sleep tracking, three on blood pressure, two on heart disease, six on covid pandemic, three on safety and six on Validations. Figure 2 shows the distribution of these studies graphically. An analysis of the keywords used in these studies indicates that most were related to smartwatches, Covid-19, and digital health. A visual representation of their distribution can be found in Fig. 3.

**Table 2:** Characteristics of studies included in the scoping review

Author/century	Study design	Participant/Subject	Aim/Objective	Result/Outcome
**Stress monitoring**				
Muhammad Ali Fauzi's et al. (2022) [1] / Norway	Experimental	15 participants	Compared three learning strategies for stress detection tasks	The experiment involved using smartwatches to collect physiological data, create classifiers, and monitor the stress levels of hospital staff in real time
Morales A et al. (2022) [2] / Brazil	Systematic Review	38 articles	The aim of the study is to develop a stress surveillance system that can help individuals manage their stress levels and improve their quality of life	The study identified several physicochemical parameters that can be used to monitor stress levels, including heart rate variation, cortisol analysis, skin conductance, body temperature, and blood volume at the wrist. The article concludes that developing a wrist wearable for stress identification using physiological and chemical sensors is challenging but possible and applicable
Muhammad Ali Fauzi's et al. (2021) [3] / Norway	Experimental	Seven machine learning methods	This study proposed a method for detecting continuous stress using single classifiers and classifier ensembles	It was found that single classifiers had the best accuracy, LR had the best precision, and NN had the best recall for stress detection
Vila G et al. (2019) [4] / France	Experimental	1604 workers	The study focuses on developing a system that monitors passengers' stress levels during air travel and provides real-time feedback to cabin crew	The study's outcome is a proof-of-concept for a real-time stress monitoring system that can improve passengers' experience during air travel
Han HJ et al. (2020) [5] / United State America (USA)	Experimental	17 participants	The study focuses on developing a system that can monitor physiological and behavioural data, including heart rate, skin conductance, and facial expressions, to estimate stress levels	The outcome of the study is a proof-of-concept for a stress monitoring system that can be used in everyday settings to improve the user's well-being
Gjoreski M et al. (2020) [6] / Slovenia	Experimental	23 participants		
Francisco de Arriba Pérez et al. (2018) [7]/ Spain	Experimental	12 Students	This study proposes a protocol to evaluate solutions based on commercial-off-the-shelf (COTS) wrist wearables to estimate student stress	The protocol was carried out in two phases: an initial laboratory-based stress induction test and a monitoring stage in the classroom while the student performs academic activities
**Movement disorders**				
Syed Mustafa Ali et al. (2021) [8] / United Kingdom (UK)	Longitudinal observational study	Fifty-three individuals	Examined the longitudinal engagement of users of a smartwatch app in people living with MLTC-M, stratifying engagement patterns by age, gender, number of disease domains and question type	This study suggests that people living with MLTC-M can use smartwatches to report multiple symptoms per day and that this data could be integrated into electronic health records to support clinical care
Amiri et al. (2017) [9] / USA	Feasibility study	12 healthy subjects aged 23 to 33 years (165 samples were collected) And two subjects aged 15 and 16	developed an Internet-of-Things (IoT) framework called WearSense that uses the sensory capabilities of modern smartwatches to detect stereotypic behaviours in children with autism	This study used a smartwatch to record motion data and develop a system to detect and monitor autistic behavioural activities. The system had an accuracy of 96.7% in detecting three autistic actions
Juan C. Torrado's et a. (2017)[10] / Spain	Experimental	Two people with autism disorder	This study focused on using smartwatches to help people with autism spectrum disorders with emotion regulation problems	They found that the smartwatch can help individuals with alexithymia and emotional dysregulation control their stress episodes triggered by various stimuli, except for the learning phase of the experiment
Stephen A et al. (2019) [11] / USA	Experimental; accelerometer data	They recruited 20 older adults	They tested whether a smartphone or smartwatch could detect whether an older adult was walking with or without an assistive device	They found that smartwatches provided much higher quality data for detecting walkers and canes compared to smartphones and that a second sensor on the hip was required for the user-generated classifiers to make the most accurate predictions
Tchuente, Franck et al. (2020) [12]/ Canada	Experimental	They recruited 30 able-bodied people (15 male, 15 female)	In this study, smartwatches are used to classify aggressive movements, which could benefit care providers in settings where people suffer from movement disorders	The kNN and ReliefF combination demonstrated that this smartwatch-based approach is a viable solution for identifying aggressive behavior and could be used by care providers in settings where people suffer from dementia or mental health disorders, where random aggressive behaviors often occur
**Sleep tracking**				
Chen et al. (2021) [13] / China	Experimental	20 subjects	In this study, ApneaDetector was developed as a smartwatch-based system to detect sleep apnoea	The study found that while the ApneaDetector accurately classified normal and sleep apnoea events, it had problems with more specific categorisations such as OSA, CSA, and hypopnoea
Chen et al. (2021) [14] / China	Experimental	20 patients	A pilot study was conducted to analyze the effectiveness of a smartwatch with seven sensors in screening for Obstructive Sleep Apnea (OSA). The study generated respiratory waveforms and detected sleep–wake states using PPG (photoplethysmography) signals	PPG-based smartwatches were more effective than simultaneous in-lab PSG or HSAT devices when screening suspected cases of OSA
Mehrabadi et al. (2020) [15] / USA	Experimental	45 healthy individuals	This study investigated the accuracy of sleep data from the Oura ring compared to medically approved actigraphy devices in 45 healthy individuals aged between 18 and 55	The results revealed that the Oura ring was significantly more accurate than the Samsung Gear Sport smartwatch in detecting heart rate, sleep, and activity parameters
**Blood pressures**				
Falter et al. (2022) [16] / Belgium	Prospective, single-arm, cross-sectional study	40 patients	Consecutive patients scheduled for 24-h ambulatory blood pressure monitoring were recruited from the cardiology outpatient clinic	This study has found that the Samsung Galaxy Watch Active 2 has an anchoring point when calibrating the device, resulting in an overestimation of lower blood pressures (BPs) and an underestimation of higher BPs. This indicates that the smartwatch is not ready for clinical usage due to its systematic bias toward a certain calibration point
Mark Tsou et al. (2021) [17] / Taiwan	Experimental	49 subjects	This study examined the applicability of smartwatches in PM2.5 health assessment	The results indicated that the elevated PM2.5 concentration was significantly associated with G-HR for low-intensity activities and marginally associated with G-HR for moderate- to high-intensity activities
Yen et al. (2022) [18] / Taiwan	A randomised controlled trial (RCT)	adults aged 20–65	This study was conducted as a single-blinded, two-arm study to test the effectiveness of a commercial smartwatch with a BP-monitoring feature	This study demonstrates that using a smartwatch with BP-monitoring features can improve blood pressure and other health parameters, increase awareness of high blood pressure, and modify related risk factors
**Heart disease**				
Bumgarner et al. (2018) [19]/ USA	A prospective, nonrandomised, and adjudicator-blinded study	100 patients	This was a non-randomized study in that the accuracy of the KB automated algorithm was evaluated by comparing its results to physician-interpreted KB rhythm strips and simultaneous ECGs	Physician interpretation of KB tracings produced by smartwatches demonstrated similar results in testing with 99% sensitivity, 83% specificity, and a K coefficient of 0.83. This suggests that the quality of KB tracings produced by smartwatches is highly reliable and accurate
Liao et al. (2022) [20] / Taiwan	Randomised	116 patients	This study evaluated the accuracy of the AF detection algorithm by obtaining ECG waveforms and PPG signals from patients undergoing AF catheter ablation while considering other arrhythmias' impact on it	Results suggest that using a longer length (25-beat) for analysing PPG data leads to higher accuracy in discriminating AF from SR compared to using only 10 beats
**Covid pandemic**				
Abbasi et al. (2020) [21] / USA		333 participants	In this study, researchers analysed data collected from 333 participants who used the DETECT smartphone app to enter symptoms and test results	The model analysed both symptoms and sensor data, and the results showed that the distinction between positive and negative cases was more accurate
Niela-Vilén et al. (2021) [22]/ Finland	A longitudinal cohort study design	38 pregnant women	This longitudinal cohort study investigated the use of an Internet of Things (IoT)-based system and smartwatch technology for monitoring pregnant women	The findings of this study showed that the pandemic-related restrictions were associated with increased heart rate variability, stress levels, decreased physical activity, and decreased sleep duration
Hunter et al. (2022) [23] / Great Britain or UK	Multicenter Observational Study	mean age of 57 years	In the study, Fitbit Charge 3 watches were given to each participant to follow up on their complications after the recovered disease	This study demonstrated that smartwatches can monitor physical activity remotely
Mishra T et al. (2020) [24] / USA	Cohort	5,262 participants	This study explored whether wearable devices could detect COVID-19 at an early, pre-symptomatic stage	It was determined that abnormal physiological events, such as elevated resting heart rate and increased heart rate relative to the number of steps, can be detected using a smartwatch at or near the time of infection
Quer et al. (2022) [25] / USA	Cohort	7298 volunteers	This study hypothesised that there are digital, objective biomarkers of reactogenicity that could be identified by detecting subtle deviations from an individual's normal resting heart rate	They demonstrated that it is possible to detect physiologic manifestations of reactogenicity to COVID-19 vaccination through individual changes in RHR
Guan G et al. (2022) [26] / USA	Cohort	1534 participants	This study uses the Garmin Vivosmart 4 smartwatch to measure heart rate and heart rate variability	This study showed that smartwatches are more accurate than patients' self-reports in predicting post-vaccination physiological conditions
**Safety**				
Lacour P et al. (2020) [27] / Germany	Single case	148 patients	This prospective observational study evaluated the potential for electromagnetic interference with CIEDs	This study shows that there is no risk of EMIs between the iPhone and CIEDs, but relatively frequent telemetry interferences do occur between the iPhone and the CIEDs
Tzeis S et al. (2021) [28] / Greece	Cohort	171 patients	This prospective, multicenter study was conducted to investigate whether the use of the latest generation smartwatches might interfere with the proper functioning of the CIED	The emission levels of the tested smartwatches and their magnetic chargers were evaluated by measuring low-frequency magnetic fields between 110 and 400 kHz
Fischer et al. (2022) [29] / Switzerland	Prospective comparative	13 different settings	This study evaluated a popular smartwatch for its ability to monitor noise levels in 13 occupational and recreational settings accurately	Smartwatch accuracy was lower in settings with rapid acoustic fluctuations but comparable to the sound level meter across different pressure levels based on SD of LAeq differences and ICC results
**Validations or evaluation**				
Pope et al. (2019) [30] / USA		21 healthy college students	This study examined four popular smartwatches' validity, measurement bias, and precision in assessing EE, average HR, and peak HR during active play	This study suggested that smartwatches should not be used as part of a systems medicine approach to health care
Shenglong et al. (2022) [31] / China		20 healthy Chinese participants	This study measured individual VE, VO2 and VCO2 using the Cosmed K5 system for a wide range of metabolic rates	The findings of this study indicate that smartwatches may have moderate validity in estimating energy expenditure for outdoor walking and running
Sarhaddi et al. (2022) [32] / Finland	Observational study	28 healthy individuals	This study evaluated the validity of the Samsung Gear Sport smartwatch in terms of HR and HRV parameters compared with a medical-grade chest ECG monitor in a 24-h continuous free-live setting monitoring	The smartwatch can accurately measure HR and HRV parameters during sleep and awake time and provide acceptable RMSSD, SDNN, LF, and HF
Brew B et al. (2022) [33] / Australia	Cross-sectional study	22 volunteer participants	This study used a threshold-based algorithm programmed for different smartwatches to detect a fall on 22 volunteer participants automatically	This study found that an algorithm programmed in commercially available smartwatches to detect induced falls had an overall sensitivity of 77% and specificity of 99%. The fall detection performance depends on the algorithm used, and the sensitivity ranges from 70 to 100% and the specificity from 80 to 100%, depending on the type of fall. In addition, they showed that the performance of a fall detection algorithm could be strongly dependent on the smartwatch model
Auepanwiriyakul et al. (2020) [34] / UK	Combined methods design	15 healthy volunteers	This study evaluated raw IMU sensor data quality, followed by a trial of the feasibility of wearable health devices in a clinical environment	In this study, wearable IMU technology, such as smartwatches and research-grade IMU Xsens, was cleaner linear acceleration signals and fewer errors than Axivity due to additional magnetometer and strap-down integration technology. This technology is viewed positively by healthcare professionals, according to this study on its acceptability among hospital patients
Varghese et al. (2021) [35] / Germany	Prospective study	400 participants	This study was a prospective study from 2018 to the end of 2021, in which they recruited and measured the hand movements of 400 participants using Apple smartwatches and smartphones. The aim was to distinguish PD from other movement disorders and healthy individuals	Results showed that smartwatches had high agreement with seismological sensor validation in measuring movement subtleties or hand-tremor amplitudes and frequencies more accurately than clinical documentation or human vision

### Stress level

As one of the leading branches of human–computer interaction, affective computing uses technology to detect a person's emotional state [29]. A stress detection system can be used for various purposes, such as monitoring the stress of drivers, detecting and alleviating the stress of passengers, monitoring the stress levels of employees, and assisting psychologists with online therapy sessions [30]. Stress detection occurs in various environments, including laboratories, hospitals, clinics, offices, schools, cars and everyday situations. When the brain receives sensory signals from the eye, nose and ear, it triggers a stress response. As a result of the stress response, heart rate increases, muscles tense, blood pressure rises, frequency of inhalation increases, blood sugar levels rise, and senses become heightened. Perceived stress is the way a person interprets and analyses stressful situations [31]. It can be assessed through regular self-reporting by individuals. Self-reported perceptions of stress can misrepresent stressful episodes, and people tend to forget stressful events, contributing to discrepancies between physiological and perceived stress levels [32]. Instruments to measure stress should be unobtrusive and non-invasive to collect data accurately. The latest technologies provide us with non-invasive and completely transparent devices to monitor stress. Heart rate variability (HRV) is one of the best-known signals for stress detection. Several features have been used in the literature to distinguish between stress and relaxation, including mean RR, mean heart rate, normalised low frequency, sympathovagal balance index (SVI) and morphological variability (MV) [33]. Combining features from smartphones and wearable devices (EDA, HR sensors) would be necessary to develop a successful stress detection system. Collecting contextual information (activity, social interaction, GPS and ambient light) about a user will also help researchers to anticipate their condition [34, 35].

Photoplethysmography (PPG) is a low-cost optical technique for measuring blood volume pulse by light absorption by blood. BVP features can be used directly or to extract heart rate variability or IBI features. A wearable physiological measurement device must provide high-quality data that is complete, relevant, timely, sufficiently detailed, appropriately presented and contains enough contextual information to facilitate decision-making and provide accurate results. Muhammad Ali Fauzi's study [36] compared three learning strategies for stress detection tasks. All three strategies used logistic regression (LR) as a machine learning model. Unlike individual learning, this learning strategy is based on a central server that combines the data and trains the integrated model. The user devices only need to perform the task of detecting stress and inferring its cause, while the server performs the tasks of feature extraction and training the model. In this study, a comparison is made between individual, centralised and federated learning for smartwatch-based stress detection. Individual learning provides higher accuracy and privacy than centralised and federated learning. The results of this study show that federated learning performs relatively mediocre in stress detection. The average accuracy was 0.8575, the average precision was 0.9892, the average recall was 0.5208, and the average F1 -measure was 0.6339. In his study, Fauzi proposed an application that could monitor the physiological signals of health professionals to detect occupational stress. The smartwatches would collect data from individual sensors, such as heart rate and skin temperature, to detect changes in physiological signals. The data would then be used to create individual classifiers and sets of classifiers to detect stress levels. In addition, the experiment found that these classifiers could be used in real-time to monitor the stress levels of hospital staff effectively.

In one interesting review study, a wearable platform on the wrist was used to detect and analyse cortisol from small amounts of sweat to study stress [35]. In some studies, the Empatica E4 wristband includes biomarkers that measure skin temperature, movement-based activity (accelerometer), electrodermal fluctuation and blood volume pulse [37–40]. Studies have used various wearable technologies, including Microsoft Band (10%), Cortiwatch (10%), ARM -Cortex4 smart wristband with DSP functionality (10%) and Empatica E4 (10%), which detects and analyses cortisol in small amounts of sweat on the wrist to detect stress [41]. A wearable device, such as a smartwatch sensor, and machine learning techniques can be used to detect stress in hospitals. However, some wearable devices are not user-friendly and uncomfortable to wear at work (e.g. devices worn on the chest, GSR sensors positioned with the finger, etc.). A study by Fauzi proposed a method for detecting continuous stress using single classifiers and classifier ensembles. This study used seven machine learning methods for stress detection, including Naive Bayes, Support Vector Machines, Neural Networks and an ensemble approach. It was found that single classifiers had the best accuracy, LR had the best precision, and NN had the best recall for stress detection. In addition, the ensemble approach performed better than all individual classifiers [37].

### Movement disorders

Several studies have shown that smartphones can track symptoms of various diseases over the long term, including chronic pain, rheumatoid arthritis, heart failure and COVID-19. It allows researchers to understand better how symptoms change over time, which can help inform treatment and management plans. The study by Syed Mustafa Ali [42] examined the longitudinal engagement of users of a smartwatch app in people living with MLTC-M, stratifying engagement patterns by age, gender, number of disease domains and question type. In the 'Watch Your Steps' study, people living with MLTC-M were asked to complete several daily and weekly questions and active tasks over 90 days. The Google Fit Research team developed the "Watch Your Steps" study app, which asked participants to complete three types of tasks: Core symptom questions, organ-specific questions and active tasks, including a sit-stand test, a walk test and a tap test. Fossil Sport smartwatches were preloaded with the study app and loaned to the participants. They were instructed to dock their watch every night for charging, and contact details for support in case of problems were included in the instruction manual. The engagement was recorded over time using longitudinal charts of daily completion rates. Fifty-three individuals with MLTC-M participated in the study. The majority were white, and the average completion rate was 45%. Most participants did not find the data collection tasks distracting, and almost all reported that the smartwatch did not interfere with their normal daily activities. They showed that using a smartwatch to collect health data is feasible and acceptable for people with MLTC-M over 90 days. This study suggests that people living with MLTC-M can use smartwatches to report multiple symptoms per day and that this data could be integrated into electronic health records to support clinical care.

Autism is a disorder with three characteristic symptoms: social development, communication and repetitive behaviours. These behaviours occur when a child tries to regulate sensory input from their environment. The study by Amiri et al. (2017) [43] developed an Internet-of-Things (IoT) framework called WearSense that uses the sensory capabilities of modern smartwatches to detect stereotypic behaviours in children with autism. They recruited 12 healthy subjects aged 23 to 33 years, from whom 165 samples were collected. They also recruited two subjects aged 15 and 16 who had been diagnosed with autism and recorded sensor data from them as they went about their regular daily routines. The subjects were asked to perform three tasks: hitting their hand in front of their face, drawing on a piece of paper and bumping their head. Participants wore a Moto360 SmartWatch protected by a 3D-printed shield that sent and stored data to a smartphone via Bluetooth. The study employed the built-in accelerometer of a smartwatch to accurately detect three distinct behaviors commonly observed in children with autism: hand flapping, painting, and sobbing. The processing component extracted 34 distinct features in each dimension of the tri-axis accelerometer, yielding a total of 102 discernible characteristics. Subsequently, several classification techniques were evaluated and compared for efficacy; ultimately demonstrating that an ensemble comprising 40 decision trees K-fold cross-validation rendered the highest level of accuracy at approximately 94.6%. This impressive degree of accuracy effectively underscores both the high caliber data captured from the smartwatch as well as commendable feature extraction methodologies implemented throughout this inquiry. Furthermore, employing a smartwatch for identifying these targeted behaviors holds significant promise in facilitating ongoing monitoring efforts pertaining to individuals who manifest autistic tendencies—thereby enabling comprehensive analysis and informed decision-making amongst parents, caregivers, and clinicians alike.

The system had an accuracy of 96.7% in detecting three autistic actions. Juan C. Torrado's [44] study focused on using smartwatches to help people with autism spectrum disorders with emotion regulation problems. It also presents and evaluates a smartwatch and smartphone system designed to accomplish these tasks. The smartwatch in this system detects anger outbursts and displays self-regulation activities previously obtained from the caregiver (smartphone). The detection is done through the smartwatch's sensor technology and involves a process of data collection, training, and evaluation. Smartwatches have a small screen and a tactile surface, so interaction is mainly through touch and sliding. There are some recent studies on text input approaches, but they limit ourselves to the simplest known interaction options: short touches and horizontal sliding. The smartwatch authoring tool has been implemented on the Android platform to make it as easy as possible for family members, caregivers, or others who are responsible for the person to use it. They used LG Watch Urbane smartwatches, a Nexus 5 smartphone, and an Android Wear interface to experiment with self-regulation strategies. They designed a simple assistance system that included linear sequences of "screens" with images, text, animations (GIFs), and positive reinforcement at the end with personalized content and a question format. They showed the results for each day and each user and enumerated the events when the self-regulation strategies were triggered. The results showed that the smartwatch helped users regain a state of calm. They found that the smartwatch can help individuals with alexithymia and emotional dysregulation control their stress episodes triggered by various stimuli, except for the learning phase of the experiment.

Clinicians and researchers rely on patient self-reports to understand the mechanisms of falls, but objective, real-world fall data are lacking. Providing clinically useful, objective measures of adherence to assistive devices could help reduce fall risk. Advances in machine learning have enabled activity recognition systems to monitor mobility in the elderly. Such systems work best when the information contains patterns that differ significantly between activities. In the study by Stephen A et al. (2019) [45], they tested whether a smartphone or smartwatch could detect whether an older adult was walking with or without an assistive device. They hypothesized that smartwatches would perform better than smartphones. They recruited 20 older adults from an adult day centre in Evanston, IL. They completed the Berg Balance Scale and Mini-Mental State Exam and provided written consent witnessed by a third person. They collected sensor data from participants who walked with and without assistive devices while completing the six-minute walk test, the 10-m walk test, and the standing and walking time tests. A physical therapist monitored participants' vital signs and provided on-call assistance to prevent falls. Participants wore an Android smartphone with a custom app that recorded triaxial accelerometer and gyroscope data at a frequency of approximately 50 Hz. Participants wore a phone and watched with a triaxial accelerometer, and data were collected throughout the session, including rest breaks. They collected enough data to train our classifiers with hundreds of samples and compared the classification accuracy of the smartphone with the smartwatch for all types of cross-validation. Fourteen older adults conducted a study in which they wore a smartphone and a smartwatch. Using accelerometer data, they trained machine learning classifiers that could predict whether a participant walked with or without an assistive device. They found that smartwatches provided much higher quality data for detecting walkers and canes compared to smartphones and that a second sensor on the hip was required for the user-generated classifiers to make the most accurate predictions.

Aggressive behavior is a prevalent issue among individuals diagnosed with dementia. Although caregivers have traditionally relied on direct observation to identify such behavior, this approach has limitations. However, advancements in technology present promising opportunities for addressing this challenge. Specifically, computer vision and human activity recognition technology, coupled with wrist-mounted inertial measurement units like smartwatches, holds great potential in accurately detecting aggressive events within healthcare and elderly-care facilities. To explore these possibilities further, F Tchuente et al. (2020) [46] conducted a study involving wearable smartwatch technology combined with machine learning techniques to classify human aggressive behavior. The research team collected data from accelerometer and gyroscope sensors embedded within Microsoft Band 2 devices. These recordings were subsequently analyzed using the Waikato environment for knowledge analysis, leveraging six distinct machine-learning classifiers along with three feature selectors. The selection process for classification models and feature selectors was based on various performance metrics, including accuracy, sensitivity, specificity, F-score, and Matthews correlation coefficient—an established measure used to evaluate predictive models' quality across imbalanced data sets. The study analyzed various classification methods and found that the k-nearest neighbors algorithm combined with the ReliefF feature selector exhibited exceptional effectiveness in differentiating aggressive and non-aggressive actions, boasting a remarkable accuracy rate of 99.6%. Additionally, this method demonstrated high sensitivity (98.4%), specificity (99.8%), precision (98.9%), F-score (0.987), and Matthews correlation coefficient (0.984). Conversely, models utilizing naïve Bayes or support vector machines performed poorly in this context. Furthermore, their findings revealed that wearing a smartwatch on the dominant wrist was the optimal approach for single-watch classification. This research successfully showcased how accelerometer and gyroscope data from smartwatches can be harnessed to identify aggressive movements with great precision effectively.

### Sleep tracking

*Sleep apnoea* is a sleep disorder in which breathing is interrupted and is associated with various health problems. The current diagnostic system for this disorder is costly, resulting in limited accessibility. Several methods have been proposed to detect sleep apnoea, but none focus on smartwatch technology. ApneaDetector is a smartwatch-based system developed by CHEN et al. (2021) [47] that uses built-in sensors to detect sleep apnoea. The system processes raw accelerometer data using signal denoising and calibration techniques to detect breathing cycles and possible apnoea events. According to their clinical study, 92% of OSA and 70% of hypopnoea events produce signal spikes that the system can detect. By removing linear trends in the accelerometer data for apnoea events using the first-order differentiation technique, spikes in normal sleep can also be identified from the calibrated data. A clinical sleep study was then conducted at Penn State Milton S. Hershey Medical Centre with 20 subjects using the ApneaDetector smartwatch to collect sensor data and evaluate different classification algorithms for sleep events. The study found that while the ApneaDetector accurately classified normal and sleep apnoea events, it had problems with more specific categorisations such as OSA, CSA and hypopnoea. However, the estimated total sleep time is acceptable for calculating the AHI value for diagnosing sleep apnoea. This work used the sensor data to calculate AHI, a standard metric for diagnosing sleep apnoea.

A pilot study used a smartwatch with seven sensors to screen for OSA by generating respiratory waveforms and detecting sleep–wake states using PPG signals. A machine learning algorithm established an initial screening model, evaluated the risk of sleep apnea, and utilized acceleration signal from the wrist for effective signal screening and abnormal scene discrimination. Participants were recruited from the Outpatient Department of Chinese PLA General Hospital. Physicians conducted a full examination on patients before testing for sleep monitoring. A detailed assessment of their sleep habits, physical condition, symptoms and complications was carried out. The study used polysomnography to compare the smartwatch against medical devices in diagnosing OSA among 20 patients. Results showed that the screening algorithm from the smartwatch is consistent with those from medical tests and had similar predictive ability compared to HSAT or PSG. In summary, PPG-based smartwatches were more effective than simultaneous in-lab PSG or HSAT devices when screening suspected cases of OSA [48].

Consumer wearables like activity trackers, smartwatches and rings can accurately measure sleep parameters with valid data collection and analysis. A study compared the Oura ring's sleep data to a medically approved actigraphy device in 45 healthy individuals aged between 18–55 years old who wore Gear Sport smartwatch, ActiGraph wristband, and Oura ring for a week. The Oura ring uses various sensors to estimate heart rate variability, respiratory rate, physical activity intensity along with acceleration and gyroscope data while wGT3X-BT by ActiGraph measures wrist acceleration in three orthogonal axes at 80 Hz for estimating sleep parameters. The study examined 4 sleep attributes using the Cole-Kripke algorithm and Troiano wear time validation. Results revealed that the Oura ring was more accurate in detecting these attributes than Samsung Gear Sport smartwatch [49].

### Blood pressures

Hypertension is a leading risk factor for heart disease, and unvalidated devices cannot be used in clinical practice. To increase the reliability of smartwatch-based BP measurements, more research is needed on both normotensive and hypertensive subjects. In Falter's study [50], consecutive patients scheduled for 24-h ambulatory blood pressure monitoring were recruited from the cardiology outpatient clinic. Measurements were taken using validated devices including an automatic cuff-based upper-arm sphygmomanometer and a Samsung Galaxy Watch Active 2 smartwatch calibrated at inclusion. Patients performed multiple measurements with both conventional BP monitors and smartwatches over a minimum of 24 h to ensure accuracy. A total of 40 patients participated in the study, The smartwatch overestimates BP up to 140 mmHg, after which it underestimates BP, illustrating the presence of proportional and differential bias. The precision of the smartwatch measurements is higher at higher BP values, while the precision of the gold standard method is higher at lower BP values. Daytime smartwatch measures were accurate for measuring blood pressure at 135/85 mmHg. The sensitivity and specificity were 84.6 and 88.9%, respectively. Blood pressure variability was higher in the ABPM measurements as compared to the smartwatch measurements, and the CV was significantly lower in the smartwatch measurements. The results of this study indicate that the smartwatch currently suffers from an anchoring point that is set when calibrating the device, resulting in an overestimation of lower BPs and an underestimation of higher BPs. The Samsung Galaxy Watch Active 2 shows a systematic bias toward a calibration point, overestimating low BPs and underestimating high BPs, and is not ready for clinical usage.

The study by Mark Tsou [51] evaluated the applicability of smartwatches in PM2.5 health assessment by evaluating whether smartwatches are good complements to certified medical devices for PM2.5 health studies, especially for developing countries. A total of 49 subjects were recruited. Each subject carried a small low-cost sensing device for personal PM2.5 and temperature monitoring for 7 consecutive days. The smartwatch employs optical HR measurement technology, and the data can be downloaded from Garmin Connect. During the same monitoring period, subjects wore smartwatches and ECG monitor devices (RootiRx, Rooti Labs Ltd.,Taipei, Taiwan) for 2 consecutive days. The activities recorded were categorized as follows: resting, commuting, working, cooking, worshipping, shopping, exercising, eating, bathing/showering, sedentary activities, and other activities. PM2.5 levels were significantly associated with heart rate (HR) in males and females aged 40 to 64 years and 65 to 75 years, respectively. The effects of PM2.5 on HR were presented as percentage changes per interquartile range (IQR) increase, and 95% confidence intervals (CI) were included. Heart rate was associated with personal PM2.5 exposure in models adjusted for subject, age, gender, body mass index, temperature, activity, and time of day. The results indicated that the elevated PM2.5 concentration was significantly associated with G-HR for low-intensity activities and marginally associated with G-HR for moderate- to high-intensity activities.

Commercial smartwatches offer a potential strategy for healthy behaviour modifications through 24-h BP, dynamic BP variability and heart rate monitoring. However, there is currently insufficient evidence to support their use in improving hypertension management. Yen [52] conducted a single-blinded, two-arm study to test the effectiveness of a commercial smartwatch with BP-monitoring feature. The study included adults aged 20–65 living in Taipei City and had an experimental group (wearing ASUS VivoWatch BP) and control group (using Mi Smart Band 3 without BP monitoring). Both groups had similar characteristics at baseline, but the experimental group showed significant improvements in DBP, SBP, resting HR, body weight, BMI, body fat, and skeletal muscle index compared to the control group. Participants' blood pressure and resting heart rate improved after 3 months of using a smartwatch with BP-monitoring feature. Their body weight, BMI, body fat and skeletal muscle index also decreased. The smartwatch increased awareness of high BP and helped modify related risk factors. However, only DBP had a significant correlation between the data from the smartwatch and sphygmomanometer.

### Heart disease

The Kardia Band is an Apple Watch accessory that can diagnose atrial fibrillation (AF) through an automatic algorithm. In a non-randomized study [53], patients with AF wore the KB before and after scheduled elective CV procedures. The accuracy of the KB automated algorithm was evaluated by comparing its results to physician interpreted KB rhythm strips and simultaneous ECGs. The study found that KB automated interpretation had high sensitivity and specificity in diagnosing AF compared to physician interpreted 12-lead ECG and KB rhythm strip. The algorithm correctly diagnosed AF with 93% sensitivity, 84% specificity, and a K coefficient of 0.77 when compared to electrophysiologist-interpreted ECGs. Physician interpretation showed similar results with 99% sensitivity, 83% specificity, and a K coefficient of 0.83 for assessing the quality of KB tracings produced by smartwatches.

PPG technology is utilized for passive and continuous monitoring of AF detection algorithms through modern wearable devices. The algorithm should have low computational cost and memory requirements to ensure superior diagnosis. A study evaluated the accuracy of the AF detection algorithm by obtaining ECG waveforms and PPG signals from patients undergoing AF catheter ablation while considering other arrhythmias' impact on it. The study included 116 patients with paroxysmal AF and 40 patients with persistent AF. Various PPG features were analyzed, including time domain and frequency domain analyses of PPI, peak height analysis of PPG, and ACF features of PPI. The majority of these features showed significant differences between the AF and SR signals in univariate analysis. Results suggest that using a longer length (25-beat) for analyzing PPG data leads to higher accuracy in discriminating AF from SR compared to using only 10 beats. Additionally, frequent PVCs/PACs can reduce the accuracy of the algorithm used for detecting AF [54].

### Covid pandemic

A recent study conducted at Scripps Research Translational Institute explored the potential of using wearable sensor data to forecast the transmission patterns of Coronavirus disease in 2019. The research team analyzed a dataset consisting of information from 333 participants who actively used the DETECT smartphone application. This application allowed individuals to input their symptoms and test results while simultaneously collecting additional biometric data such as heart rate and sleep patterns through commercially available wearable devices. By incorporating symptom-based indicators and sensor-generated data into their analytical model, researchers achieved significantly higher accuracy levels in distinguishing between positive and negative cases compared to models solely relying on symptoms alone [55].

Technology has enabled continuous monitoring of personal health parameters, such as stress, physical activity, and sleep, during pregnancy. In order to address issues of this nature, the use of an Internet of Things (IoT)-based system and smartwatch technology for the monitoring of pregnant women was investigated in a longitudinal cohort study design. The study involved participants wearing smartwatches continuously from early pregnancy through three months after the birth of the child. Participation in the study was restricted to Finnish-speaking women carrying singletons during the 12th—15th week of pregnancy. A total of 38 pregnant women were monitored during the COVID-19 outbreak in Finland for a period of eight weeks. Monitoring system based on IoT was developed to collect signals from Samsung Gear Sport smartwatches every two hours for 12 min. PPG signals were used to extract parameters related to heart rate and heart rate variability. During the study, the Samsung watch was used to measure physical activity and sleep, and TST and WASO were calculated for each night. To analyze the data, the Statsmodel Python package was used, and the dependent variables were measurements of HRV, physical activity, and sleep. The findings of this study showed that the pandemic-related restrictions were associated with increased heart rate variability, stress levels, decreased physical activity, and decreased sleep duration. Pregnant women can benefit from the use of Internet of Things (IoT) technologies in monitoring their daily patterns of well-being [56].

The occurrence of lung damage and potential post-treatment injuries is a significant concern in the context of the widespread covid-19 outbreak. Consequently, both patients affected by this disease and medical personnel involved in their care may benefit from monitoring potential complications even after recovery. A valuable instrument that can assist with such surveillance is the utilization of smartwatches. In the study conducted by Hunter [57], Fitbit Charge 3 watches were given to each participant along with their anonymized study reference ID numbers. The data from a smartwatch was extracted, including the daily step count and the daily resting heart rate. They defined smartwatch use as wearing the watch for a minimum of one month. Participants were recruited across sites in South East England, with a mean age of 57 years, 74% being White, and 54% having at least one comorbid condition. Within three months of discharge, the mean step count of the entire cohort increased by 37%. At 3 months and 12 months following discharge, the participants' mean heart rates were reduced by approximately 7% and 13%, respectively. While a considerable number of participants did not use smartwatches on a regular basis, this study demonstrated that smartwatches are capable of monitoring physical activity remotely.

Early detection of infectious diseases is crucial in order to reduce disease spread by enhancing self-isolation and enabling early treatment. Despite this, current diagnostic methods involve sampling and nucleic acid-based tests require a substantial amount of time and money. Testing methods currently available are unlikely to identify presymptomatic carriers. Therefore, real-time detection of cases is imperative. It is possible to detect COVID-19 infection well before symptoms appear, using smartwatches and other wearable devices. In order to accomplish this, a real-time heart rate monitoring algorithm was developed to detect early stages of infection. In this study, they explored whether wearable devices could detect COVID-19 at an early, pre-symptomatic stage. In this manner, a method for potentially detecting the onset of illness in real time, in the early stages, was developed. In this study, two methods were developed with the objective of detecting aberrant physiology in humans: the RHR difference method (RHR-Diff) and the heart rate over steps anomaly detection method (HROS-AD). The study enrolled 5,262 participants who responded to surveys regarding their illness, diagnosis and symptom dates, illness severity, and type of symptoms. Among these, 4,642 reported wearing a smartwatch, of which 3,325 wore Fitbits, 984 wore Apple watches, 428 wore Garmin devices, and the remaining wore other devices. Upon enrollment on REDCap, participants were directed to download the MyPHD app, which collects wearable device data in a de-identified and encrypted manner. The data collected by wearable devices included heart rate, steps, and sleep. The data were retrieved at intervals of 15 s, 1 min, and sleep stage. Metadata and symptom surveys were downloaded from the participants and processed using a custom-written R and Python script. Accordingly, 88% and 100% of individuals with symptom onset or diagnosis dates showed elevated signals prior to or at the time of onset or diagnosis, respectively. It was determined that the increased RHR signal had a specific relationship to COVID-19 by analyzing 15 cases of non-COVID-19 illness. According to the findings, elevated heart rates that occur before illness can be used as a general indicator of respiratory illness. Infection with COVID-19 alters sleep and activity patterns, which can be monitored using a wearable device. The duration of sleep and the number of steps decrease at the onset of the outlying RHR-Diff signal that is associated with COVID-19 illness. With this prototype, 63% of COVID-19 infections were detectable with an alarming frequency of 0.66 per month in healthy individuals. It was determined that abnormal physiological events, such as elevated resting heart rate and increased heart rate relative to number of steps, can be detected using a smartwatch at or near the time of infection [58].

Three vaccines are currently authorized and distributed in the United States to prevent the spread of COVID-19. Although there is substantial variability in individuals' immune response to vaccines, the CDC V-safe program found that the majority of individuals reported some systemic side effects after the second dose. A recent study found a relationship between reactogenicity symptoms after vaccination and a humoral immune response. In the study conducted by Quer et al. (2022) [59] collected daily wearable sensor data from 7298 volunteers who received at least one dose of the COVID-19 vaccine. They hypothesized that there are digital, objective biomarkers of reactogenicity that could be identified by detecting subtle deviations from an individual's normal resting heart rate. In the DETECT study, 7,298 participants received at least one mRNA vaccination. Of these, 5674 (78%) participants contributed adequate data to evaluate changes in activity and sleep, respectively. They observed that the average RHR increased the day following vaccination, reaching a peak on day 2 and not returning to baseline until day 4 and 6, respectively. The majority of participants experienced an increase from their normal RHR. They explored several participant and vaccine characteristics that could impact immune response, and found that women experienced higher RHR changes with respect to baseline in the 5 days following vaccination after the first dose only. In contrast, RHR responses vary by age, with individuals age 40 having the greatest increase in RHR. Although a direct comparison is not possible, changes comparable to the ones observed after the second dose of the Johnson & Johnson vaccine were detected in their cohort. After adjusting for potential confounding factors, prior COVID-19 infection was independently associated with a higher RHR increase after the first dose, and female sex was independently associated with a higher RHR increase after the first dose. After adjusting for age, device, vaccine type, and prior COVID-19 infection, they observed higher RHR increases from Apple devices on average, but not after the first dose. The first dose of the vaccine had minimal effect on activity and sleep, but the second dose caused a significant decrease in activity and an increase in sleep, which returned to baseline by day 2. They demonstrated that it is possible to detect physiologic manifestations of reactogenicity to COVID-19 vaccination through individual changes in RHR. This provides a potential novel mechanism to identify individuals with either a suboptimal or exaggerated immune response to a vaccine. Similarly, a study using the Garmin Vivosmart 4 smartwatch to measure heart rate and heart rate variability showed that smartwatches are more accurate than patients' self-reports in predicting post-vaccination physiological conditions [60].

### Safety

The safety of wearable technologies in clinical settings is a critical factor influencing the advancement and growth of these tools. The integrity and accuracy of patient clinical measurements obtained through these devices must be established to avoid any potential risks that could compromise the health conditions of individuals under medical care. Since smartwatches are primarily designed for commercial purposes, examining the safety considerations associated with utilizing such wearable tools for patients becomes an essential aspect explored within this study.

High-frequency electromagnetic fields are produced by mobile phones and smartwatches. As such, these devices have the potential to affect human health, although the issue is still under debate. Hence, in order to examine the safety of these devices, a prospective observational study was conducted in order to evaluate the potential for electromagnetic interference with CIEDs. It was conducted using a popular smartphone and smartwatch, which provided real-time monitoring and printing of intracardiac electrograms, marker channels and a 3-lead electrocardiogram. In total, 1,352 testing procedures were conducted on 148 patients for cardiac implantable electronic devices, which included 51 pacemakers, 5 cardiac resynchronization therapy pacemakers, 46 inverter defibrillators, 43 cardiac resynchronization therapy defibrillators, and 3 implantable loop recorders. EMIs were observed between the iPhone and an implanted dual-chamber pacemaker in 1 patient, but not between the Apple Watch and the CIED. In connecting mode, an iPhone placed directly over the generator was observed to cause EMI, which resulted in marker channel assignment being lost, as well as EGM loss and noise in the ventricular marker channels. Moreover, this study shows that there is no risk of EMIs between the iPhone and CIEDs, but relatively frequent telemetry interferences do occur between the iPhone and the CIEDs [61].

There is the potential for electromagnetic interference to adversely affect cardiac implantable electronic devices (CIEDs), such as pacemakers, implantable cardioverter-defibrillators, cardioversion-defibrillators, and cardiac resynchronization therapy pacemakers and defibrillators. Considering their inductive charging functionality, smartwatches could be a significant source of electromagnetic interference (EMI) due to their ability to transfer power wirelessly over distances up to four centimeters (QiTM). The magnetic components in smartwatches emit electromagnetic fields that can interfere with patient monitoring systems and defibrillators. A prospective, multicenter study was conducted to investigate whether the use of the latest generation smartwatches might interfere with the proper functioning of the CIED. The study participants were 171 patients who received CIEDs and presented to two centers in Athens, Greece, for routine follow-up between March and November 2019. Their tests were conducted on two smartwatches of the latest generation for potential EMI as well as on their magnetic chargers. The ECG recording was meticulously analyzed to identify atrial and ventricular pacing inhibition, asynchronous ventricular pacing, rapid ventricular pacing, and asynchronous pacing. The emission levels of the tested smartwatches and their magnetic chargers were evaluated by measuring low-frequency magnetic fields between 110 and 400 kHz. Each smartwatch was activated and measured separately, first directly in contact with the probe, then at a distance of 10 cm and 20 cm [62].

Hearing loss is caused by repeated exposure to loud noises, as well as metabolic disorders, hypertension, abnormal sleep patterns, occupational accidents, tinnitus, and a reduction in cognitive abilities. In situations in which noise levels are high, people should move away from the source or wear hearing protection. However, noise-induced hearing loss (NIHL) may occur slowly and remain undetected for a considerable period of time. Smartphones can be used to measure noise levels, but there are several limitations. Wearable devices such as smartwatches can overcome smartphone limitations because they are worn on the wrist. In the study conducted by Fischer [63], a popular smartwatch was evaluated for its ability to accurately monitor noise levels in 13 occupational and recreational settings. The results showed that the smartwatch and a sound level meter used as a reference were in excellent agreement. It was found that the music club (52% of the measurement) emitted the most hazardous noise levels, followed by construction sites (24%), housekeeping (20%), and streets (4%). The sound level meter and smartwatch measurements are offset by an average of 0.5 dBA (SD of 1.8 dBA), which indicates the smartwatch underestimates the sound level. This study compared noise levels measured by a smartwatch and sound level meter. Smartwatch accuracy was lower in settings with rapid acoustic fluctuations, but comparable to the sound level meter across different pressure levels based on SD of LAeq differences and ICC results.

### Validation or evaluation

Despite the fact that smartwatches' health metrics have generally demonstrated high levels of moderate-to-strong validity, limitations in the current literature exist, including that EE and HR data validity of smartwatches has been found to be less valid in free-living PA assessments, and that most previous validation studies have utilized various Fitbit or Jawbone models. Accordingly, Pope's [64] study examined the validity, measurement bias, and precision of four popular smartwatches in assessing EE, average HR, and peak HR during active play. The participants for this study were 21 healthy college students from a large metropolitan Midwest U.S. university. They were ages 18 to 35, had a body mass index of 18.5 and performed high-intensity exercise that elicited EE > 300 kcalories for each session. ActiGraph GT3X + -BT accelerometers were used in conjunction with an ActiGraph HR strap attached to the chest for the measurement. For EE data collection, a 1-s epoch was employed, with the following empirically derived cut-points (in counts/minute): light PA: 0—2690; moderate PA: 2691—6166; vigorous PA: 6167—9642; and very VPA: q9643. ActiGraph HR straps mounted on the chest were moistened with water 2 cm below the nipple, and smartwatches were preprogrammed for a 20-min exercise session. They downloaded EE and HR data from the ActiGraph using ActiLife 6.13, and identical data was collected for each smartwatch. Smartwatches demonstrated moderate-to-good precision for average HR and peak HR measurements with approximately the same accuracy as an ActiGraph. EE assessment with the ActiGraph GT3X was found to be valid in comparison to doubly labelled water. However, this tool is not considered the gold standard for EE assessment. In spite of the fact that smartwatches can provide moderate validity when assessing average and peak HR, smartwatch EE assessments are less reliable. Hence, this study suggested that smartwatches should not be used as part of a systems medicine approach to health care.

There has been a growing trend for fitness toward the use of wearable technology over the past few years. A variation in EE estimation accuracy may occur depending on the type and intensity of activities, and algorithms may not take into account the type and intensity of physical activity or the posture of the body. Validating EE estimations requires consideration of the intended use of the device, as well as an assessment of the accuracy of EE estimations made by young adults while walking and running outdoors. Accordingly, Shenglong et al. (2022) [65] recruited twenty healthy Chinese participants from the campus of the local university for their study. They measured individual VE, VO2 and VCO2 using the Cosmed K5 system for a wide range of metabolic rates. EE was calculated using the ratio between inhaled oxygen and exhaled carbon dioxide. Three smartwatches were examined for validity: Apple Watch Series 6, Garmin Fenix 6, and Huawei GT 2e. Photoplethysmography was used to determine heart rate and GPS was used to determine distance and speed while walking or running outdoors. Data for this study was collected during one visit. Subjects had not consumed food, coffee, tea, or other stimulants, did any vigorous physical activity, or consumed alcohol during 24 h prior to measurements. EE data were taken from the K5 breath-by-breath and summed for each exercise session separately. The EE estimates were obtained directly from the watches. The validity of the smartwatches was determined by several statistical tests, including paired sample t-tests, mean absolute percentage errors (MAPE), and Intraclass Correlation Coefficient (ICC). Excellent, good, moderate, and low agreement thresholds were defined as ICC values of ont, goo). The Apple Watch Series 6 had the highest energy expenditure, followed by the Garmin FENIX 6, and the Huawei Watch GT 2e. GF could likely provide better EE estimates for the outdoor walking than running, and EE tends to be overestimated at lower pace and underestimated at higher pace. Smartwatch EE overestimated EE versus the K5 during outdoor walking and running, and the energy cost of running was overestimated by 24.4% and 21.8%, respectively. This may result in the ineffectiveness of a weight loss program. The findings of this study indicate that smartwatches may have moderate validity in estimating energy expenditure for outdoor walking and running.

Physiological parameters such as heart rate and heart rate variability provide insight into cardiovascular and autonomic nervous system dysfunction as well as mental, physiological, and sleep-related stress. Noninvasive methods for HR and HRV monitoring include Electrocardiography (ECG) and Photoplethysmography (PPG). Studies evaluated the accuracy of HRV parameters extracted from wristbands and smart-watches including Apple Watch, Empatica E4, Microsoft band 2, and the Wavelet wristband against medical-grade ECG devices. They showed that motion artifacts highly affect the reliability of HRV parameters. In the study conducted by Sarhaddi et al. (2022) [66] evaluated the validity of the Samsung Gear Sport smartwatch in terms of HR and HRV parameters compared with a medical-grade chest ECG monitor in a 24-h continuous free-live setting monitoring. The study included 28 healthy individuals. They collected data using two wearable devices and self-report and background questionnaires. The participants wore a Samsung Gear Sport smartwatch and a Shimmer3 ECG device, and logged their sleep and non-wear time. The Shimmer3 ECG device was used to measure ECG as the gold standard method in their assessment. Also, they developed a customized data collection application for the Samsung Gear Sport watch to collect 16 min of PPG signals every 30 min continuously. They used a deep-learning-based method for PPG peak detection, which is enabled by a dilated Convolutional Neural Networks (CNN) architecture. Also, they developed a two-round peak detection algorithm to locate peaks in ECG signals that obtains higher accuracy in comparison with Pan-Tompkins and Hamilton algorithms. The correlation between the HR and HRV parameters of the smartwatch and Shimmer3 was high (positive), and the LF/HF ratio value showed a moderately positive relationship. The regression analysis was used to compare the accuracy of the extracted parameters from the Samsung smartwatch against the reference ECG. The HR, AVNN, and pNN50 parameters showed good agreement with the ideal lines, but the other HRV parameters showed relatively diverging lines. The Samsung smartwatch underestimates AVNN values, but overestimates other parameters. The Samsung smartwatch and reference ECG showed a high positive correlation between the AVNN and HR values, a moderate positive correlation between the HR and the other HRV parameters, and low positive correlations between the LF, HF, and LF/HF ratio. The results indicate that the accuracy is highest when the watch's parameters are equal to the golden standard values. They validated the accuracy of HR and HRV parameters extracted from PPG signals collected by the Samsung smartwatch during sleep and awake time using short-term HRV analysis. The Samsung smartwatch underestimates HR, SDNN, LF, and LF/HF ratio but overestimates AVNN during sleep time and awake time. Moreover, the watch underestimates RMSSD, pNN50, and HF during sleep time, although it overestimates these parameters during awake time. In concluded this smartwatch can accurately measure HR and HRV parameters during sleep and awake time, and provide acceptable RMSSD, SDNN, LF, and HF.

Falling occurs more frequently with age, and staying on the floor for a prolonged period of time after a fall can have serious consequences, such as hospitalization, a decline in activities of daily living, or a placement in a long-term care facility. There is an increasing availability of assistive technologies, such as call alarm systems and personal emergency response systems. However, consumers are not always able to use these technologies due to difficulties activating these systems. Fall detection can be performed using an app on a smartwatch. The study conducted by Brew B et al. (2022) [17] aimed to address these issues. This study used a threshold-based algorithm programmed for different smartwatches to automatically detect a fall on 22 volunteer participants. 12 participants were wearing two smartwatches, model A and B, and 10 participants were wearing only one smartwatch, model C, on one wrist. In three phases, the algorithm collected acceleration data and the time of the fall: "prefall", "induced fall" (8 falls around five minutes), and "postfall" (walking back from the crash mat to the area to remove the smartwatches). This study found that an algorithm programmed in commercially available smartwatches to detect induced falls had an overall sensitivity of 77% and specificity of 99%. The fall detection performance depends on the algorithm used, and the sensitivity ranges from 70 to 100% and the specificity from 80 to 100% depending on the type of fall. In addition, they showed the performance of a fall detection algorithm could be strongly dependent on the smartwatch model [17].

Sensors that measure movement in the body have the potential to revolutionize how clinical status and wellbeing are measured in daily healthcare. Physical disabilities can be described, quantified, and monitored in these instruments, deteriorations can be detected, and treatment responses can be monitored. In a research or clinical setting, consumer smartwatches cannot be used due to their unknown data quality. A smartwatch IMU's accuracy and precision can be evaluated by measuring both absolute errors and correlations with ground truth values. In the study conducted by Auepanwiriyakul [67], raw IMU sensor data quality was evaluated, followed by a trial of the feasibility of wearable health devices in a clinical environment. Using the Apple Watch Series 3 and 5 IMUs, they compared the inertial accuracy of the consumer smartwatches with two well-known research- and clinical-grade IMU sensors and a gold-standard test for human movement assessment utilizing optical motion tracking. The researchers recruited 15 healthy volunteers and developed a WatchOS App that collected triaxial acceleration and triaxial angular velocity data in real time on Apple Watches at a frequency of 100 Hz in order to record and extract inertial data from Apple Watches. As the base for the experiment, they used an Apple Watch Series 3 and adhered the OptiTrack markers pad to the base with the aid of double-sided tape. Apple Watches demonstrated weak to moderate R2 agreement with OptiTrack. Apple Watch Series 3 and 5 had strong R2 agreement with each other for acceleration and angular velocity. They also had strong agreement with Xsens MTw Awinda. In this study the consumer smartwatches (Apple Watch Series 3 and 5) and research-grade IMU Xsens achieved cleaner linear acceleration signals and lower errors than Axivity, perhaps due to the additional magnetometer and strap down integration (SDI) technology. According to their study on the acceptability of wearable IMU technology by hospital patients and healthcare professionals, this technology is viewed favourably.

Consumer wearables equipped with multisensor technology are an effective means of monitoring objective movement patterns. Various systems have potential for diagnosing PD by analyzing voice, hand movements, gait, facial expressions, eye movements and balance. However, caution should be exercised when interpreting reported accuracies as the models were trained on low sample sizes (n < 100) regarding PD. Varghese et al. (2021) [68] conducted a prospective study from 2018 to the end of 2021, in which they recruited and measured hand movements of 400 participants using Apple smartwatches and smartphones. The aim was to distinguish PD from other movement disorders and healthy individuals. They compared acceleration amplitudes and tremor frequencies utilizing a seismometer and high-precision shaker with different machine learning models trained for classification performance assessment. Participants wore two smartwatches during a 15-min neurological examination, designed by movement disorder experts. A Trillium Compact seismometer measured ground velocity as part of the study. A shaker table experiment was conducted to validate the method using two Apple watches and a Trillium Compact seismometer. The z-axis accuracy of the method was tested with tremor-typical frequencies and amplitudes, resulting in 43 measurements performed on different days. The watches were connected to iPhones via Bluetooth while data from the seismometer were collected on a digitizer that it was attached to. Each measurement lasted for 20 s for the watches, which were mounted on the shaker table along with a decoupled platform for placing the seismometer. The study utilized various models including SVM, CatBoost, MLP and DL to extract features from acceleration data using Keras and Tensorflow. Results showed that smartwatches had high agreement with seismological sensor validation in measuring movement subtleties or hand-tremor amplitudes and frequencies more accurately than clinical documentation or human vision.

## Discussion

The fast-paced advancements in technology have given rise to a new category of patients called e-patients. These individuals rely on various technological tools to monitor and track the progression of their diseases [4]. Scholarly works, such as those by Asad et al. (2019) [69], Masters et al. (2017) [4], Loda et al. (2019) [70], and Herrmann-Werner et al. (2019) [71] highlight the need for medical curriculum integration that enhances communication skills and resolves the challenges associated with engaging e-patients. The studies suggest training medical students on utilizing e-patient knowledge in healthcare delivery, advising patients regarding credible online sources, assessing website credibility strategies, and employing blended-learning teaching methods to improve students' competence when dealing with this unique patient population. Therefore, it becomes crucial for clinical students to possess the necessary skills required for effective engagement with these technologically-savvy individuals. This can only be achieved through comprehensive training provided by educational institutions. Wearable technologies like smartwatches are increasingly popular in healthcare due to their potential to optimize practices and promote healthier habits [72]. However, integrating these devices presents challenges like seamless integration with clinical workflows and efficient data management [73, 74]. Medical students, who use smartwatches, serve as role models for patients, making it crucial for future healthcare professionals to possess technical proficiency and a thorough understanding of these technologies.

Thanks to technological advancements, smartwatches and wearable technology are rising in the diagnosis and symptom reporting field [67, 75]. Researchers have conducted studies to assess these technologies' effectiveness, with encouraging outcomes. An investigation revealed that smartwatches and wearables are accurate when identifying initial signs of health issues like irregularities in heart rate [64]. Moreover, users can receive valuable feedback through this technology. Additionally, researchers discovered that such devices play a crucial role in detecting potential health hazards at an early stage before they escalate into critical conditions [52]. Another study examined how wearables can monitor patients with chronic conditions. The study discovered that these devices have the capability to perceive variations in essential indicators such as pulse rate and blood pressure, notifying medical professionals of possible issues [64]. This could prove especially advantageous for individuals unable to communicate effectively with their healthcare providers or are susceptible to abrupt fluctuations in health.

Recent studies have shown that smartwatches can be incredibly useful for various purposes, including motion sickness, heart health, work stress and timely diagnosis of COVID-19 [55, 57]. The watch has the capability to assess respiratory rate, levels of oxygen saturation, and body temperature. This functionality provides the potential to detect the virus early on [55]. However, it should be noted that this method does not possess the ability to differentiate between infections caused specifically by SARS-COV-2 versus other viruses. Nevertheless, its use could lead to identifying diseases arising from diverse infectious agents and potentially forecasting disease severity as well as symptom manifestation [76]. Disease detection using wearable devices offers many advantages over traditional testing methods, such as no testing infrastructure, materials or personnel, passive testing and high-resolution continuous screening to allow follow-up testing and self-isolation. Although smartwatches are increasing and digital technology was widespread during the COVID-19 pandemic, few studies have used smartwatches for rehabilitation, empowerment or patient engagement in rehabilitation [57, 77]. Moreover, the smartwatch can track heart rate and identify any abnormalities, which could serve as an initial indication of potential cardiovascular issues [10, 66]. Additionally, employers can utilise smartwatches to monitor employee stress levels in work settings and access valuable information that aids in providing necessary support for their workforce.

Pairing the smartwatches utilized by the patient with additional smart wearable devices that can be employed by both the patient's companions and even healthcare professionals in the intensive care unit holds immense potential to transform it into an invaluable information tool. This confluence of technological advancements plays a pivotal role in diligently monitoring and tracking crucial symptoms exhibited by the patient [75]. Smartwatches incorporate advanced technology to deliver precise and up-to-date data, enabling medical professionals to comprehend their patients' health conditions. Significantly, individuals have the convenience of monitoring their vital signs and other pertinent health information. They can securely send this information to their caregivers or healthcare professionals to improve the effectiveness of monitoring their state [75]. This ultimately improves the quality of patient care and enhances understanding of the patient's overall wellness. Furthermore, secure transmission of this data helps expedite diagnosis and treatment processes by reducing time requirements [78, 79]. Consequently, patients receive faster and more efficient treatment as a result. Additionally, these smartwatches serve as reminders for patients to take medication promptly, ensuring an added level of comfort and safety in managing their health conditions. It is important to note that smartwatches' functionality heavily relies on their algorithmic operations and program design alongside the specific type utilized in healthcare settings [53].

Examining the viability of smartwatches as dependable tools necessitates thoroughly considering safety. Previous studies, such as Elvis, have emphasized the significance of user safety in determining the sustained intention to utilize smartwatch applications [80]. In line with this, Lee has developed an effective tool to assess smartwatch quality from users' perspectives by evaluating factors like usability, functionality, and, most notably, safety concerns [81]. Moreover, Hong's research in 2022 reveals the positive impact that both safety and convenience can have on individuals' motivation to incorporate smartwatches into their lifestyles [82]. Alongside assessing their ability to measure clinical data accurately, this research also delves into the Safety concerns surrounding patients' usage of these devices. The findings from multiple studies highlight that smartwatches demonstrate satisfactory levels of safety when used by individuals [61–63].

The world of smartwatches has significantly advanced in recent years. However, most of these watches have been developed for convenience rather than to help with medical diagnosis [75]. Recent studies indicate that smartwatches' potential in detecting medical symptoms may be limited due to issues with precision and accuracy. The study conducted by Varghese revealed that although smartwatches were capable of capturing subtle tremor signs in Parkinson's disease, there were noticeable differences in amplitude and frequency when compared to a seismometer [68]. Similarly, the research carried out by Falter showcased insufficient accuracy in smartwatch-based blood pressure measurements, showing a systematic bias towards a calibration point [50]. Antognoli, on the other hand, emphasized the necessity of metrological validation for wearable devices and determined that while commercial smartwatches offered certain precision and accuracy for heart rate and blood pressure measurements, some degree of bias was present [83]. Moreover, Poli's investigation from 2022 also examined the precise nature of blood pressure tracking using smartwatches; however, it suggested lower data quality as compared to medical-grade instruments despite achieving acceptable levels of precision and accuracy [84]. Hence, the research indicates that currently available smartwatches may not meet the necessary standards for precise and accurate detection of medical symptoms. These findings raise concerns about the effectiveness of smartwatches in accurately monitoring and detecting health conditions.

The trustworthiness of smartwatches is a subject of concern due to the opaqueness surrounding the algorithms used for detecting and analyzing users' health information. These algorithms are kept from the general public, which introduces a level of uncertainty regarding their accuracy and reliability [79]. Furthermore, research studies have pointed out that different brands of smartwatches yield varying results when diagnosing clinical symptoms, sometimes even diverging from those provided by traditional medical devices [84–86]. Recent studies have examined the accuracy and precision of various smartwatch models compared to medical-grade pulse oximeters. Windisch discovered that although the Apple Watch Series 6 generally demonstrated good agreement with medical-grade devices, outliers were still reported, reaching up to a 15% difference [86]. Jiang, on the other hand, conducted a comparative analysis of different smartwatches and found that the Apple Watch Series 7 performed closest to the reference standard while the Garmin Venu 2 s deviated furthest from it [85]. These findings suggest that while some smartwatches showcase favorable alignment with medical-grade pulse oximeters regarding accuracy assessments, occasional outliers as well as discrepancies remain present due to variations among different models and contexts considered during evaluations.

Given the crucial nature of healthcare decisions, relying entirely on these watches' information becomes increasingly challenging. This could pose a notable challenge in certain situations where precise diagnosis and treatment are crucial. This study underscores both the significance and pressing need for incorporating smartwatch technologies into health services. It is essential to acknowledge that although smartwatches can serve as valuable tools for monitoring health and overall wellness, they should not be solely relied upon for diagnosing illnesses in most instances [87]. Hence, we recommend that leading companies in healthcare technology venture into this domain, ensuring that they provide smartwatches equipped with dependable and efficient mechanisms for monitoring individuals' well-being. This calls for the development of medical watches and the creation of AI-hospital assistants.

Smartwatches have the potential to revolutionise healthcare by providing patients with easier access to their health data. They also increase the acceptance of long-term home monitoring [88]. However, smartwatches in healthcare also raise problems. One problem is that smartwatches focus on aggregating biomedical data and do not take a holistic view of the patient [78]. The over-reliance on smartwatches and the fewer face-to-face doctor visits lead to a violation of the principle of non-maleficence [78].

With the increasing availability of 6G technology, AI and medical data, the potential of predictive analytics is becoming more real. This opportunity allows us to develop algorithms that have incredibly high diagnostic power. Using these datasets, we can identify patterns and trends that can be used to predict future health conditions and make informed medical decisions [79]. Smartwatches has a broad reach and can be significantly improved, but its security and credit ratio still needs to be assessed. This review has attempted to highlight the strengths and weaknesses of some smartwatches in the context of diagnosis and validation; however, further research is needed.

## Conclusion

Advancements in technology have led to the rise of e-patients who rely on technology to monitor and track their diseases. Medical curriculum integration is crucial for enhancing communication skills and addressing challenges associated with engaging e-patients. Wearable technologies like smartwatches are increasingly popular in healthcare, but integrating them presents challenges. The review highlights the relationship between smartwatches and their efficiency in health systems. Smartwatches can be effective in diagnosing and reporting symptoms, detecting potential health hazards early, and monitoring patients with chronic conditions. However, safety concerns need to be considered, as most smartwatches have been developed for convenience rather than medical diagnosis. The trustworthiness of smartwatches in healthcare is also a concern due to the opaqueness of their algorithms and varying results in diagnosing clinical symptoms. Instead, there is an increasing demand for a clinical assistant that relies on AI within the health network. This type of assistant would be able to offer more precise and dependable data while also being better equipped to handle the intricacies involved in healthcare. Given these findings, additional investigation will be imperative to fully comprehend and exploit the potential of smartwatches in healthcare and develop more sophisticated and efficient clinical assistants that utilise AI technology.

## Data Availability

Considering that the study is a review, no data are available.
